# Initial psychometric properties of the Parental Stress Scale examined using a sample of Russian mothers

**DOI:** 10.3389/fpsyg.2023.1202401

**Published:** 2023-09-05

**Authors:** Alexandra A. Bochaver, Diana R. Akhmedjanova, Roksana M. Bayramyan, Elizaveta V. Fomicheva

**Affiliations:** Centre for Modern Childhood Studies, Institute of Education, HSE University, Moscow, Russia

**Keywords:** parental stress, parental well-being, parent–child relationships, parenting, stress measure

## Abstract

Parental stress is defined as a personal response to stressors associated with being a parent. In recent studies, parental stress has been viewed as a component of normative parenting. The purpose of this study was to collect initial evidence of the construct validity and reliability of the Russian version of the 18-item Parental Stress Scale (PSS) using a sample of mothers of Russian primary school students. The results are the first wave of a longitudinal study. Mothers (*n* = 900) of fourth-grade students participated in the study and filled out an online survey. The exploratory and confirmatory factor analyses of the PSS on the Russian mothers indicated two factors: parental stress and parental satisfaction, with good estimates of reliability. The PERMA-Profiler questionnaire was used to examine the convergent and divergent validity of the PSS. The results revealed significant correlations between parental stress and satisfaction and different aspects of well-being among the respondents. The initial investigation of this Russian adaptation of the PSS provides evidence of its reliability and validity. Despite the limitations and the need for further research, this version of the PSS can be recommended for use in studies on modern parenting as well as in psychological support, education, and development of programs promoting positive parent–child relationships by targeting parental needs.

## 1. Introduction

The construct of parental stress was introduced by Selye (1978) and has been actively researched since. Extensive research on the subject in the 1980s–1990s produced a nuanced understanding of stress (Lazarus, 1999). Currently, parental stress is defined as a personal response to stressors associated with being a parent and executing a parenting role (Abidin et al., 2022). It is an intensely negative reaction toward oneself and/or the child that occurs when a caregiver feels overwhelmed or lacking in the skills and resources required to fulfill the requirements of their parental role and has difficulties adapting to it (Rivas et al., 2021). Parental stress may manifest in all parents to varying degrees (Deater-Deckard and Scarr, 1996) and may be considered a psychological cost of being a parent (Deater-Deckard, 1998). It differs conceptually from other life stressors, such as financial problems or negative life events, although they are frequently related (Rivas et al., 2021).

Studies on parental stress have been conducted since the 1980s, initially focusing on clinical and high-risk populations and medical contexts to examine the stress experienced by parents (Abidin, 1982; Abidin and Wilfong, 1989). Gradually, in the 1990s, the view of parental stress shifted toward normalization. Research has shown that parenting stress might be a normative process that affects every parent, although its previous definition included parents’ perceptions of their children’s behavior and their feelings of incompetence in parenting (Deater-Deckard and Scarr, 1996). As Crnic and Greenberg (1990) wrote, “all parents have some experience with being nagged or whined at, settling arguments between siblings, repeatedly cleaning up their children’s messes, as well as a myriad of other possible everyday events of a similar nature. Although any one of these events may have little significance in and of itself, their cumulative impact over a day, several days, or longer may represent a meaningful stressor for a parent” (1628).

Crnic and Greenberg (1990) used the term “hassles” to describe the irritating demands that characterize the everyday transactions of parents with their environment. These hassles may be infrequent and situationally determined, or repetitive and stable.

Research studies have typically identified child and parental components of parental stress. The main child-related stressors are the daily routines associated with caring for children and the child’s undesirable behaviors. Parent-related stress can be caused by both objective factors (e.g., reduced time for sleep and leisure) and subjective ones (e.g., the perception of oneself as an incompetent parent; Crnic and Greenberg, 1990). Family composition and transitions such as separation or incarceration have also been found to be related to higher stress (Webster-Stratton, 1990; Barbot et al., 2014; Maguire, 2015; Steele et al., 2016; Gil-Rivas et al., 2017; Louie et al., 2017). A higher level of stress is typical for parents who see their children as difficult and demanding, or who perceive themselves as ineffective parents. Contradictory results have been obtained as to whether mothers or fathers tend to be more stressed (Deater-Deckard and Scarr, 1996; Deater-Deckard, 1998; Yeh, 2002; Oelofsen and Richardson, 2006; Alzawad et al., 2021; Perez-Brena et al., 2021; Gómez-Ortiz et al., 2023).

Parental stress has been associated with poor, harsh, neglectful, or abusive parenting, and was suggested to be a predictor of negative social adjustment in children. However, individual differences and parental behavior were discovered to be mediators between parental stress and child outcomes (Deater-Deckard, 1998). Factors relating to both the parent and the child contribute to parenting stress and are affected by it in a complex transactional process with consequences for the well-being of both parties (Crnic and Ross, 2017).

Parental experience is complicated, and recent studies within the social-ecological framework have demonstrated reciprocal relationships between parental and child behavior (Rivas et al., 2021). For example, children with a diminished sense of security display anger or distress, increasing parental stress and thereby creating a chaotic family environment. This then contributes to children’s behavioral problems and feelings of powerlessness, which lead to low self-esteem and high anxiety. Levels of parental stress are related to individual child differences and developmental maladjustment (Louie et al., 2017), particularly depressive symptoms, autism spectrum disorders (ASD), and attention deficit hyperactivity disorder (ADHD; Thomason et al., 2014; van Steijn et al., 2014; Mackler et al., 2015; Stone et al., 2016; Barroso et al., 2018). Parental stress could also lead to child maltreatment and adverse childhood experiences, such as witnessing or experiencing domestic violence or receiving verbal and emotional abuse (Calvano et al., 2022; Geprägs et al., 2023). Therefore, it seems appropriate to consider parental stress more as a symptom indicating various problems, such as a family’s low SES, parent’s psychiatric disorders, illnesses, and difficult behavior of the child, than as a separate phenomenon.

In Russia, parental stress has not been studied intensively, and the articles dedicated to it have mostly only been published in the last few years (Savenysheva et al., 2019). However, despite this recent increased interest in investigating parental stress, there are few measures available. The Parental Stress Index has been validated on a Russian sample (Vasilenko et al., 2021) but is not yet available for free. To assess parental distress, questionnaires on daily stressors (Petrash et al., 2018) and parental burnout (Efimova, 2013) can be used. Our study is aimed at addressing the lack of research on parental stress and of valid and reliable measures, specifically by validating the Parental Stress Scale (PSS; Berry and Jones, 1995).

### 1.1. Parental stress scale

There are several reliable tools to measure parental stress (Holly et al., 2019). Since the 1980s, the Parenting Stress Index (PSI), a 120-item self-reported measure, has been widely used in clinical and research contexts. However, the most compact and psychometrically sound survey available is the Parental Stress Scale (PSS; Berry and Jones, 1995). PSS was developed as an alternative to PSI and was based on a transactional model of stress, where parental stress was conceptualized as a bidirectional interaction between parents and children. According to Louie, “A transactional model of parenting stress was novel; it challenged the dominant view in parenting at the time that focused on the impact of parents on children” (Louie et al., 2017, p. 361). Due to the bidirectional and complicated nature of parent–child relationships, this 18-item questionnaire includes items measuring not only opportunity costs and limitations on personal resources (stress and lack of control by parents) but also rewarding aspects of parenting, such as fulfillment and personal growth, that contribute to the parenting experience.

Although several studies support the initial factor structure and reliability of PSS, there is no consensus regarding which and how many of the original 18 items should be included, nor is there a robust factor structure with satisfactory reliability. Berry and Jones (1995) collected data from a heterogeneous sample of mothers and fathers of both neurotypical, normally developing children and children receiving school-based or outpatient services for emotional and/or behavioral problems. The initial psychometric research on PSS identified four factors: Parental Rewards (Items 1, 5, 6, 7, 8, 18), Parental Stressors (Items 3, 9, 10, 11, 12, 16), Lack of Control (Items 14, 15, 16), and Parental Satisfaction (Items 13, 17, 18). Two items (16 and 18) yielded significant loadings on two of the factors, whereas two items (2 and 4) failed to load on any of the four factors. These findings “support the dichotomy of the parenting experience and the theoretical bases of the Parental Stress Scale” (Berry and Jones, 1995, p. 470). Changes to item number and wording and to the response scale were made in subsequent studies. The samples in these studies included parents of children of different ages and different health statuses, parents with health issues, stepparents, grandparents, and people from various cultural backgrounds. However, a person’s background can influence how questions are understood and may have impacted the results. Louie et al. (2017) recommended changing the initial wording of Item 2 because of its repeated ambiguity. In Iran, an adaptation of PSS given to a sample of 500 mothers of premature infants resulted in three factors: parenting stress, mother-infant turbulent interaction, and parental expectations (Habibpour et al., 2018). In Indonesia, statistical analysis of responses from 449 parents of children aged 3–12 years revealed a two-dimensional structure among 15 of the PSS items; the other three items were removed (Kumalasari et al., 2022). In Portugal, a study of 3,842 parents of 3–10-year-old children supported the original four-factor structure of PSS (Algarvio et al., 2018). Data from Korea sampled 160 parents of children with ADHD and demonstrated two sub-factors, namely parental stress and parental satisfaction, for 11 of the items; Items 2–5, 7, 8, and 11 were deleted (Park et al., 2021). In Norway, data from a sample of 1,096 parents of one-year-old children revealed a two-dimensional structure of parental stressors and a lack of rewards across 13 PSS items (Items 1, 2, 4, 15, and 18 were removed; Nærde and Sommer Hukkelberg, 2020). In the Danish version of PSS, validated on 1,110 mothers of children aged 0 to 12 months, Items 2 and 11 were eliminated. The remaining items did not make up a unidimensional scale but rather two subscales: a nine-item scale measuring parental stress and a seven-item scale measuring lack of parental satisfaction (Pontoppidan et al., 2018).

These various PSS adaptations for use in different countries are characterized by different factor structures and varying sets of items. It is therefore important to test the scale on a sample of Russian mothers. The present study examined the psychometric properties of the Russian version of PSS and used complementary exploratory and confirmatory factor analyses to investigate the underlying factor structure of the PSS items. This work follows from that of other authors engaged in developing adaptations of PSS in different countries (Habibpour et al., 2018; Nærde and Sommer Hukkelberg, 2020; Park et al., 2021; Kumalasari et al., 2022).

### 1.2. Validity and reliability

The validation of PSS is framed in terms of the unified validity framework articulated by Messick (1995) and Hubley and Zumbo (2011), in which validity is defined as “an overall evaluative judgment of the degree to which empirical evidence and theoretical rationales support the adequacy and appropriateness of interpretations and actions on the basis of test scores or other modes of assessment” (741). Since validity is dependent on the reliability of scores (American Educational Research Association, 2014), we examined the accuracy and consistency of the scores as well. Accordingly, this paper evaluates three types of validity evidence for PSS: (1) construct validity; (2) convergent and divergent validity; and (3) reliability. The purpose of this study was to establish initial evidence for the validity and reliability of the PSS on a sample of Russian mothers as part of an ongoing longitudinal study.

## 2. Materials and methods

### 2.1. Participants and procedure

The sample comprised 900 mothers whose children (9–10 years old) were attending the fourth grade in schools in and around Nizhny Novgorod, Russia. The mothers’ ages ranged from 24 to 56 years old (Mean = 38.08, SD = 5.46). Most of the mothers had a bachelor’s degree or higher (73%).

The current validation study is part of the research project “Longitudinal Study of Factors Related to School Failure.” After receiving approval from HSE University’s Ethics Committee (#19), the data collection took place online with participants recruited from 40 public schools. Before filling out the surveys, parents were informed about the purpose of the study and signed online consent forms.

### 2.2. Instruments

The participants completed the following questionnaires:

1. The Parental Stress Scale, which includes 18 items and measures the levels of parental stress (Louie et al., 2017). Eight items are reversed, describing positive parenting experience. The original paper suggested four subscales: Parental Rewards [e.g., “I enjoy spending time with my child(ren)”], Parental Stressors (e.g., “I feel overwhelmed by the responsibility of being a parent”), Lack of Control (e.g., “Having children has meant having too few choices and too little control over my life”), and Parental Satisfaction (e.g., “I am satisfied as a parent”; Berry and Jones, 1995). Two items did not relate to any scale (2 and 4). The original response scale ranged from 1 (strongly disagree) to 5 (strongly agree), with a midpoint of 3 (undecided). In this study, the intermediate option was removed. Respondents were offered a response scale from 1 (strongly disagree) to 4 (strongly agree) for greater parental response accuracy.
  As the first step of the validation process, the PSS items were translated into Russian by an expert in psychology. Two other experts in psychology and education independently checked the Russian translation. The discrepancies in wording were resolved during the experts’ discussion with the translators and psychometricians involved in the project, including the authors. As the final step, the Russian translation of the items was back-translated into English independently by the first and second authors of the manuscript, both of whom have native-like fluency in English. See Appendix Table 1 for further details.
2. The PERMA-Profiler, the Russian adaptation of which measures five pillars of well-being (Seligman, 2011; Butler and Kern, 2016; Isaeva et al., 2022). The original survey includes 15 items across five subscales of well-being: Positive Emotion (e.g., “In general, how often do you feel joyful?”), Engagement (e.g., “How often do you become absorbed in what you are doing?”), Relationships (e.g., “To what extent do you receive help and support from others when you need it?”), Meaning (e.g., “In general, to what extent do you feel that what you do in your life is valuable and worthwhile?”), and Accomplishment (e.g., “How much of the time do you feel you are making progress toward accomplishing your goals?”). It includes eight additional items assessing Negative Emotion, Loneliness, and Physical Health which were removed from this study due to their weak factor loadings in the primary source and its Russian adaptation. The response scale in the original and in the Russian adaptation ranges from 0 (never) to 10 (always). However, for this study, we modified the response scale so that it ranged from 1 (never) to 5 (almost always). This decision was made to simplify the choices for the respondents and make the responses more interpretable. We chose the PERMA-Profiler because there is evidence of its validity and reliability for the comprehensive measurement of well-being (Butler and Kern, 2016). This scale has previously been used in conjunction with PSS to develop a tool for evaluating positive parenthood in Greece (Kyriazos and Stalikas, 2019). The reliability indices on a sample of Russian mothers (*n* = 900) indicated high estimates (ωh = 0.76, ωt = 0.95).

### 2.3. Data analysis

The data analyses were conducted in R. The *likert* package was used for the descriptive statistics and summaries (Bryer and Speerschneider, 2022). To examine the factor structure, EFA analysis was performed in the *psychometric* package (Fletcher, 2022), and CFA analysis in *lavaan* (Rosseel et al., 2023). The *psych* package (Revelle, 2023) was used to run Pearson *r* correlation analysis and identify McDonald’s omega reliability estimates. The missing data analyses were conducted using the *mice* (van Buuren et al., 2022) and *VIM* (Templ et al., 2022) packages.

## 3. Results

### 3.1. Missing data

The initial dataset of 1,071 responses was checked for missing data values and the proportion of mothers and fathers. The Pearson’s chi-squared test generated large *p*-values, which suggested that there was no association between missing information on the PSS and PERMA-Profiler questionnaires and the observed values of parental sex or income. The results indicated that the missingness mechanism was not systematic for the variables considered, and missing values were possibly missing completely at random. After removing the responses with missing data and the responses of 68 fathers due to the small sample size, the final sample included only complete observations provided by mothers (*n* = 900).

### 3.2. Exploratory factor analysis

Based on the results of PSS validation studies in different countries and the lack of a reliable factor structure, we decided to reexamine the internal structure of PSS, like Nærde and Sommer Hukkelberg (2020). We randomly split the sample (*n* = 900) into equal parts and conducted Exploratory factor analysis (EFA; *n* = 450) and CFA (*n* = 450). The EFA was conducted on the original 18 items, and CFA verified the PSS structure proposed by EFA. Factor analyses contribute to validity evidence by verifying that the latent structure of the survey fits the items, and by providing a parsimonious model to establish internal consistency (reliability) and criterion and construct validity (Boateng et al., 2018).

Before conducting the EFA, the data correlations and assumptions of factorability and sphericity were checked. The inter-item correlations indicated small to medium positive and negative correlations among items (from −0.06 to 0.74). As expected, items within the same domains were more highly correlated with each other than with items from other domains. The negative correlations resulted from the reverse-scored items in the dataset. The Kaiser Meyer Olkin (KMO) factor adequacy overall estimate was 0.91, and the estimates for each item ranged from 0.86 to 0.93. This suggested that factor analysis could be performed, as KMO estimates equal to or larger than 0.60 are considered adequate for conducting factor analysis (Dziuban and Shirkey, 1974). The Bartlett test of sphericity also suggested that it was appropriate to conduct a factor analysis, χ^2^(153) = 4858.93, *p* < 0.001.

The factor structure, based on eigenvalues and scree plots of the principal axis factor analysis, suggested a three-factor model. A separate parallel analysis suggested two factors, which were analyzed using oblique rotation. The two-factor model showed an acceptable model fit. Therefore, the factor loadings made conceptual sense and suggested two subscales, parental stress and parental satisfaction, with eight items in each (Table 1). Since Items 3 and 4 had the lowest factor loadings, we decided not to include them in the final version of the survey.

**Table 1:** Reliabilities and item level estimates for the adapted PSS (*n* = 450).

		McDonald’s omega_h_	McDonald’s omega_t_	Mean (SD)	Item total correlation	ITC if item is dropped
	Parental stress scale	0.49	0.89			
	Parental stress	0.83	0.93			
1.	I feel stressed and depressed because of the responsibility of being a parent.			1.4 (0.81)	0.83	0.78
2.	Having children has led to limited choice and control over my life.			1.6 (0.91)	0.83	0.78
3.	It is difficult for me to combine different responsibilities because of the child (children).			1.8 (0.86)	0.79	0.72
4.	The birth of my child (children) had a negative impact on my financial well-being.			1.6 (0.83)	0.81	0.75
5.	If I had to go through this again, maybe I would decide not to have children.			1.3 (0.78)	0.76	0.69
6.	I am often embarrassed or nervous because of the behavior of my child (children).			1.8 (0.87)	0.77	0.69
7.	Having a child (children) has limited my personal time and freedom in my life.			1.9 (0.96)	0.76	0.67
8.	My child (children) is (are) the main source of stress in my life.			1.7 (0.92)	0.75	0.66
	Parental satisfaction	0.75	0.9			
9.	I like to spend time with my child (children).			3.7 (0.46)	0.77	0.69
10.	I like being a parent.			3.8 (0.42)	0.76	0.68
11.	I feel a strong attachment to my child (children).			3.7 (0.52)	0.73	0.63
12.	I think my child (my children) is (are) wonderful.			3.8 (0.47)	0.64	0.53
13.	I will do anything for my child (children), if necessary.			3.8 (0.46)	0.65	0.54
14.	I feel that we have a close, trusting relationship with my child (children).			3.5 (0.55)	0.72	0.60
15.	I am more confident and optimistic about the future because I have a child (children).			3.6 (0.61)	0.70	0.57
16.	I am happy with myself as a parent.			3.4 (0.64)	0.66	0.50

### 3.3. Confirmatory factor analysis

**Figure 1:**
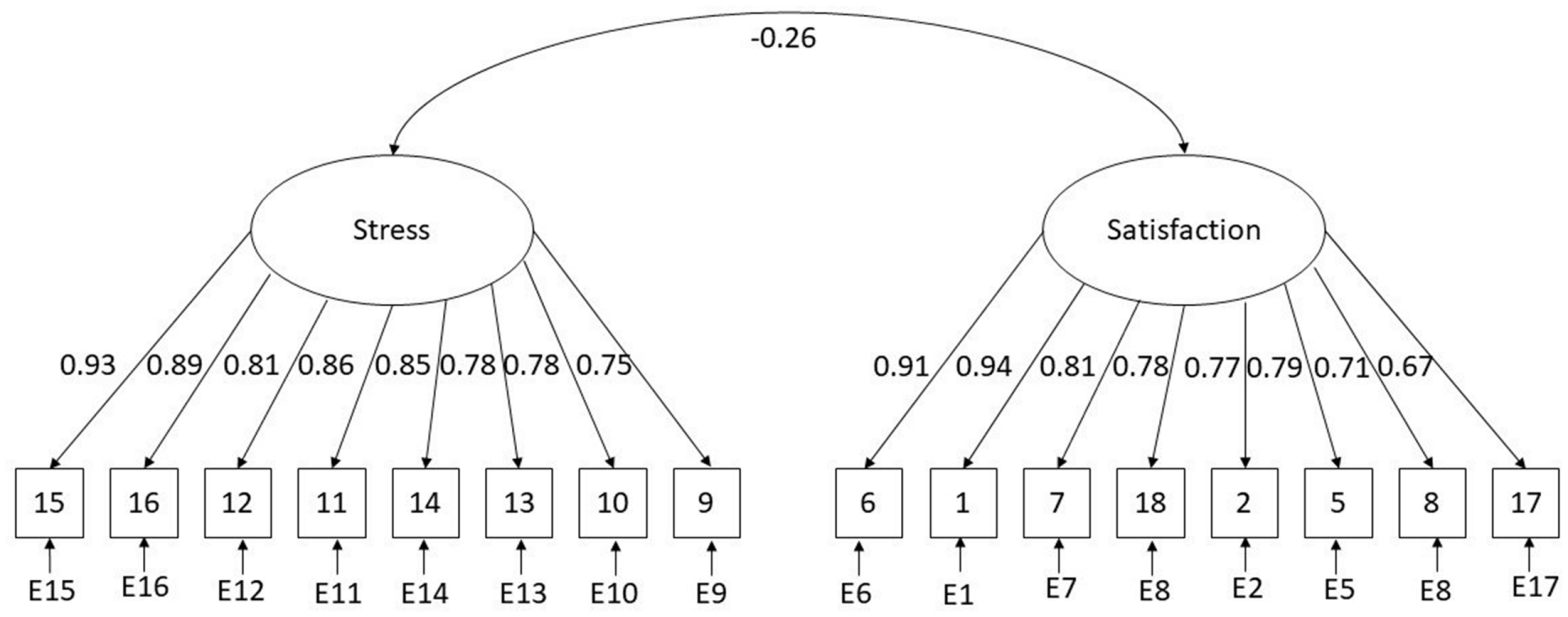
The final two-factor model of the Adapted PSS (*n* = 450).

The confirmatory factor analysis (CFA) analysis was conducted on the second half of the sample (*n* = 450) to examine the two-factor structure. The diagonally-weighted least squares (DWLS) estimator was used to estimate the model parameters due to the ordinal nature of the PSS. The CFA indicated a good model fit, χ^2^(103) = 236.04, *p* < 0.000, CFI = 0.99, TLI = 0.99, RMSEA = 0.05, SRMR = 0.06. The estimates of factor loadings are reported in Figure 1 and item-level statistics in Table 1. The χ^2^/df coefficient resulted in 2.29.

### 3.4. Convergent and divergent validity

Since establishing convergent validity requires measuring the same construct using different methods and instruments (American Educational Research Association, 2014), and we did not measure parental stress/ parental satisfaction using another scale, this paper focuses on the evidence of convergent and divergent validity using PERMA-Profiler, assessing different aspects of well-being.

The correlations between all five PERMA-Profiler subscales of well-being had statistically significant positive correlations with the subscale of parental satisfaction (from 0.09 to 0.24, Table 2), which provides some evidence of convergent validity. The correlations between the subscales of Positive Emotion, Relationships, Meaning, and Accomplishment had statistically significant negative correlations with the parental stress subscale (from −0.16 to −0.22), which is evidence of divergent validity. These results provide initial evidence of convergent and divergent validity but should be interpreted with caution, since PERMA-Profiler does not measure parental stress or parental satisfaction.

**Table 2:** Correlations among Subscales of PERMA-Profiler and PSS (*n* = 900).

	Positive emotion	Engagement	Relationships	Meaning	Accomplishment	Stress	Satisfaction
Positive emotion	1						
Engagement	0.59^***^	1					
Relationships	0.62^***^	0.50^***^	1				
Meaning	0.63^***^	0.52^***^	0.69^***^	1			
Accomplishment	0.61^***^	0.56^***^	0.60^***^	0.77^***^	1		
Stress	−0.16^***^	−0.05	−0.18^***^	−0.22^***^	−0.16^***^	1	
Satisfaction	0.15^***^	0.09^**^	0.19^***^	0.24^***^	0.18^***^	−0.09^**^	1
Mean (SD)	4.3 (0.52)	4.0 (0.6)	4.28 (0.64)	4.4 (0.60)	4.28 (0.56)	1.61 (0.67)	3.67 (0.42)

*** *p <* 0.001.

** *p <* 0.01.

### 3.5. Reliability

The reliability analysis was performed by estimating McDonald’s omega since it is a better reliability estimate than Cronbach’s alpha (Deng and Chan, 2017). The final model of the adapted PSS survey consisted of two subscales of parental stress and parental satisfaction. The reliability indices across two scales were high: parental stress (ω_h_ = 0.83, ω_t_ = 0.93) and parental satisfaction (ω_h_ = 0.75, ω_t_ = 0.9). The whole scale resulted in a hierarchical omega of 0.49, which suggests that it should consist of two distinct factors.

## 4. Discussion

This paper reports on the initial psychometric properties of the Russian adaptation of PSS, which includes 16 items and two scales. There has previously been no reliable, convenient, and short instrument with which to assess levels of parental stress in the Russian population, so this tool could potentially fill this ga PSS can be used for research in the areas of developmental, family, clinical, and educational psychology as part of the process of designing prevention programs and psychological counseling for parents.

The data analysis of the Russian version of PSS provided evidence of acceptable reliability and convergent and divergent validity. It also showed this adaptation to include two factors. This result confirms the unstable factor structure of the questionnaire and corresponds with the results assessing PSS adaptations in different countries, in which either two (Pontoppidan et al., 2018; Nærde and Sommer Hukkelberg, 2020; Park et al., 2021; Kumalasari et al., 2022), three (Habibpour et al., 2018), or four (Algarvio et al., 2018) factors were obtained. The small yet significant associations between the PSS and PERMA-Profiler scales support the conceptual closeness, but not the similarity, of their constructs. This finding is important due to the need for more specific instruments to measure parenting experiences, rather than a general, multidimensional sense of well-being.

Forty years ago, the suggested psychological intervention goals for optimizing parental stress focused on stress reduction for parents with a high level of anxiety and stress augmentation for parents with low levels of stress, in order to increase sensitivity and commitment toward their children (Abidin, 1982). Later, parental stress was perceived as a uniquely negative characteristic, although normative. As a result, practitioners started developing diverse ways to help parents cope with it.

Different protective factors against parental stress have been discovered in studies, such as family values, social support within the family (Miranda et al., 2019; Lo et al., 2023), and co-parenting alliances (Delvecchio et al., 2015). Parental stress reduction is now considered a common and relevant goal of preventive and rehabilitative parenting programs (van Steijn et al., 2014; Rivas et al., 2021; Bauch et al., 2022; Nurlaila et al., 2023). Different informational campaigns, psychological programs, and recommendations have been developed to help parents understand and deal with their own stress and that of their children. They are aimed at anger management, communication skills, awareness, time management, and other skills. The study of factors that contribute to and prevent parental stress in Russian parents is still in an early stage, although it is an important and promising area of research. In this way, our adapted PSS opens opportunities for expanding knowledge in this area.

While this study presents a short yet psychometrically sound instrument, it has some limitations. These included the homogeneity of the sample, due to the similarity of the children’s ages and only mothers’ participation; the limited set of instruments; and the collection of data in only one region of Russia. To expand the knowledge of parental stress in Russian families, further research is needed to analyze the convergent and divergent validity using more instruments and with a more diverse sample of parents of younger and older children, mothers and fathers, and people from multiple regions. In addition, future validity studies should examine intended and unintended personal and social consequences of score use and interpretation of the adapted PSS (Hubley and Zumbo, 2011). Also, given the unstable factor structure of PSS across validation studies, future work should examine parental response processes, focusing on item interpretations, testing settings and time, cultural and personal values, and other possible extraneous variables contributing to the parental responses to PSS.

## 5. Conclusion

This paper reports the initial psychometric properties of the Russian version of PSS. The initial results provide enough evidence to use PSS to measure the positive and negative aspects of parenting in Russia. The adapted scale includes 16 items across two scales: parental stress and parental satisfaction. This tool widely extends the opportunities for research in the field of family and developmental psychology in Russian-speaking populations. It can be used both to study the specifics of parental stress in different social contexts or regions of the same country and for cross-cultural comparisons. Hence, data obtained from PSS can be used to develop intervention programs aimed at decreasing home violence and child abuse, reducing parental stress, and increasing the quality of parent–child relationships.

## Data availability statement

The raw data supporting the conclusions of this article will be made available by the authors, without undue reservation.

## Ethics statement

The studies involving humans were approved by Committee on Interuniversity Surveys and Ethical Assessment of Empirical Research of the HSE University. The studies were conducted in accordance with the local legislation and institutional requirements. The participants provided their written informed consent to participate in this study.

## Author contributions

AB developed the main idea of the paper, wrote the first draft of the manuscript, and contributed to the manuscript revisions. DA contributed to the study’s conception and the first draft of the manuscript, performed the statistical analysis and contributed to the manuscript revisions. RB collected the data, organized the database, contributed to the study’s conception and design and the statistical analysis. EF contributed to the organizing the database and the statistical analysis. All authors contributed to the article and approved the submitted version.

## Funding

This study was conducted with support from the Basic Research Program of HSE University.

## Conflict of interest

The authors declare that the research was conducted in the absence of any commercial or financial relationships that could be construed as a potential conflict of interest.

## Publisher’s note

All claims expressed in this article are solely those of the authors and do not necessarily represent those of their affiliated organizations, or those of the publisher, the editors and the reviewers. Any product that may be evaluated in this article, or claim that may be made by its manufacturer, is not guaranteed or endorsed by the publisher.
