# Supplementary material for: Initial psychometric properties of the Parental Stress Scale examined using a sample of Russian mothers
Source: Front Psychol. 2023 Sep 5;14:1202401. doi: 10.3389/fpsyg.2023.1202401 (PMC10507722; doi:10.3389/fpsyg.2023.1202401)
Supplement: Supplementary file 1 [file Table_1.docx]

**Appendix A.**

**Table 1**

Original, Translated, and Back Translated Items of the PSS

| № | Original items | Russian translation | Back translation |
| --- | --- | --- | --- |
|  | I am happy in my role as a parent. | Мне нравится быть родителем | I like being a parent |
|  | There is little or nothing I wouldn’t do for my child(ren) if it was necessary. | Я сделаю все ради своего ребенка (своих детей), если это необходимо | I will do anything for my child (children), if necessary |
|  | *Caring for my child(ren) sometimes takes more time and energy than I have to give.* | *Воспитание и забота о моем ребенке (моих детях) занимает больше времени и энергии, чем у меня есть* | *Raising and caring for my child (children) takes more time and energy than I have* |
|  | *I sometimes worry whether I am doing enough for my child(ren).* | *Иногда я беспокоюсь, достаточно ли я делаю для своего ребенка (детей)* | *Sometimes I worry if I'm doing enough for my child (children)* |
|  | I feel close to my child(ren). | Я чувствую, что у нас близкие, доверительные отношения с моим ребенком (моими детьми) | I feel that we have a close, trusting relationship with my child (children) |
|  | I enjoy spending time with my child(ren). | Мне нравится проводить время со своим ребенком (своими детьми) | I like to spend time with my child (children) |
|  | My child(ren) is (are) an important source of affection for me. | Я чувствую сильную привязанность к своему ребенку (детям) | I feel a strong attachment to my child (children) |
|  | Having children gives me a more certain and optimistic view for the future. | Я более уверенно и оптимистично смотрю в будущее, потому что у меня есть ребенок (дети) | I am more confident and optimistic about the future because I have a child (children) |
|  | The major source of stress in my life is my child(ren). | Мой ребенок (мои дети) являются основным источником стресса в моей жизни | My child (children) is (are) the main source of stress in my life |
|  | Having children leaves little time and flexibility in my life. | Наличие ребенка (детей) ограничило личное время и свободу в моей жизни | Having a child (children) has limited my personal time and freedom in my life |
|  | Having children has been a financial burden. | Рождение ребенка (детей) отрицательно сказалось на моем финансовом благосостоянии | The birth of a child (children) had a negative impact on my financial well-being |
|  | It is difficult to balance different responsibilities because of my child(ren). | Мне сложно совмещать разные обязанности из-за ребенка (детей) | It is difficult for me to combine different responsibilities because of the child (children) |
|  | The behavior of my child(ren) is often embarrassing or stressful to me. | Я часто смущаюсь или нервничаю из-за поведения моего ребенка (моих детей) | I am often embarrassed or nervous because of the behavior of my child (children) |
|  | If I had it to do over again, I might decide not to have children. | Если бы мне пришлось пройти через это снова, возможно, я бы решил(-а) не заводить детей | If I had to go through this again, maybe I would decide not to have children |
|  | I feel overwhelmed by the responsibility of being a parent. | Я чувствую напряжение и подавленность из-за ответственности быть родителем | I feel stressed and depressed because of the responsibility of being a parent |
|  | Having children has meant having too few choices and too little control over my life. | Рождение детей привело к ограничению выбора и контроля над моей жизнью | Having children has led to limited choice and control over my life |
|  | I am satisfied as a parent. | Я доволен(-льна) собой в качестве родителя | I am happy with myself as a parent |
|  | I find my child(ren) enjoyable. | Я считаю своего ребенка (своих детей) замечательным(-и) | I think my child (children) is (are) wonderful |

*Note:* Items 3 and 4 in italics did not load onto the two factors and were excluded from the adapted Russian version of the PSS.
